# MicroRNA-376c-3p Facilitates Human Hepatocellular Carcinoma Progression via Repressing AT-Rich Interaction Domain 2: Erratum

**DOI:** 10.7150/jca.94900

**Published:** 2024-02-09

**Authors:** Yuan Wang, Weiping Chang, Wanli Chang, Xiaowei Chang, Song Zhai, Guoying Pan, Shuangsuo Dang

**Affiliations:** 1 Department of Infectious Diseases, the Second Affiliated Hospital of Xi'an Jiaotong University, 157 Xiwu Road, Xi'an 710004, China; 2 Department of General Surgery, the First Affiliated Hospital of Xi'an Medical University, 48 Fenghao West Road, Xi'an 710077, China

We regret that the original version of our paper unfortunately contained incorrect representative images. The wrong images were placed in Figures 3, 5A, 5D and 6E when choosing representative images from very large amount of data. The correct version of the Figures 3, 5A, 5D and 6E appears below. The authors confirm that the corrections made in this erratum do not affect the original conclusions. All the authors of the paper have agreed to this correction. The authors apologize for any inconvenience that the errors may have caused.

## Figures and Tables

**Figure 3:**
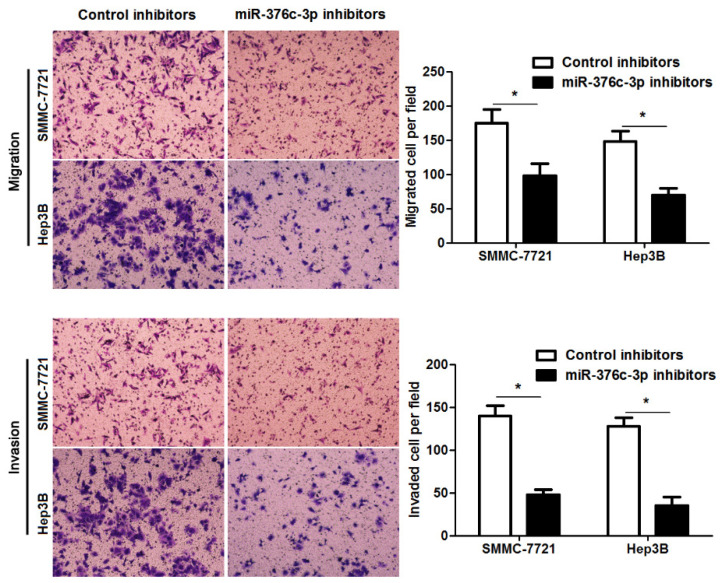
**miR-376c-3p knockdown suppresses migration and invasion of HCC cells.** SMMC-7721 and Hep3B cells were transfected with miR-376c-3p inhibitors or control inhibitors. Transwell assay indicated that miR-376c-3p knockdown significantly decreased the number of migrated and invaded HCC cells. n= five fields of three independent repeats, *P<0.05 by t test.

**Figure 5:**
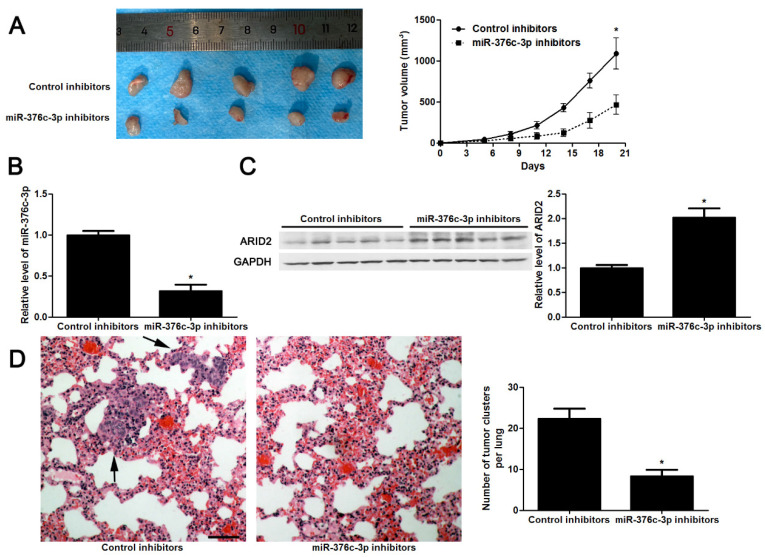
**Knockdown of miR-376c-3p inhibits HCC growth and metastasis *in vivo*.** (A) Hep3B cells that were transfected with control inhibitors or miR-376c-3p inhibitors were implanted into the right flank of the mice via subcutaneous injection (n=5 per group). the results indicated that miR-376c-3p knockdown led to HCC growth restriction in mice. *P<0.05 by ANOVA. Scale bar: 1 cm. (B) qRT-PCR analysis of miR-376c-3p expression in tumor tissues harvested from mice. n=5, *P<0.05 by t test. (C) Immunoblotting analysis of the expression of ARID2 protein in xenograft tissues arising from mice. n=5, *P<0.05 by t test. (D) Hep3B cells that were transfected with control inhibitors or miR-376c-3p inhibitors were implanted into nude mice via tail vein injection (n=5 per group). miR-376c-3p reduced the lung metastases of HCC in mice. *P<0.05 by t test. Black arrows indicated lung metastases. Scale bar: 50μm.

**Figure 6:**
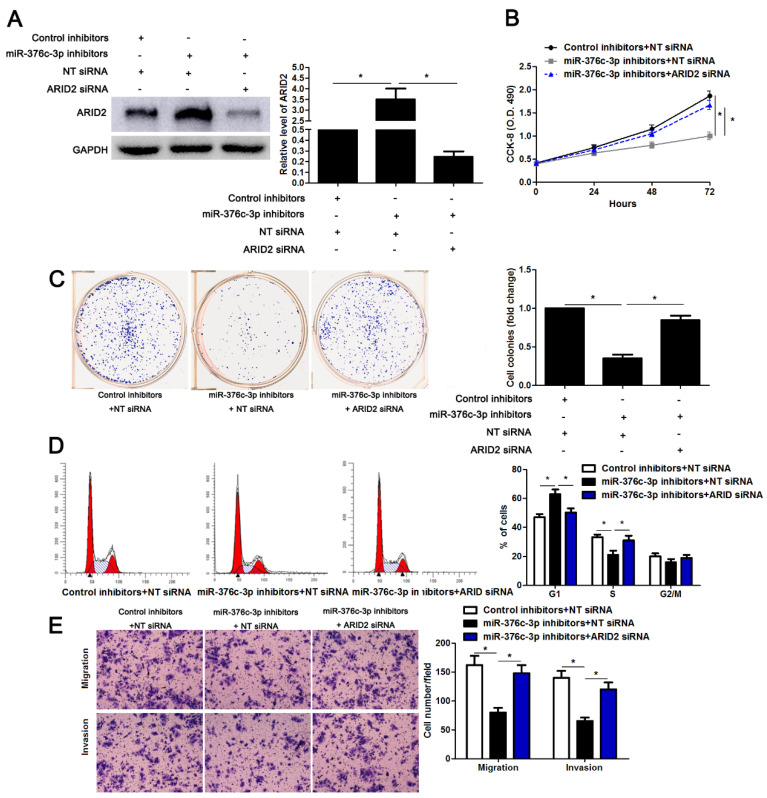
**ARID2 silencing abolishes the effect of miR-376c-3p knockdown in HCC cells.** (A) miR-376c-3p inhibitors and ARID2 siRNA were co-transfected into Hep3B cells. The protein level of ARID2 was determined using immunoblotting. n=three independent repeats, *P<0.05 by ANOVA. (B) miR-376c-3p knockdown suppressed proliferation of Hep3B cells, and ARID2 silencing subsequently promoted cell proliferation. n=three independent repeats, *P<0.05 by ANOVA. (C) ARID2 silencing increased the number of cell colonies in miR-376c-3p silenced Hep3B cells. n=three independent repeats, *P<0.05 by ANOVA. (D) miR-376c-3p knockdown led to G1 arrest, and ARID2 silencing subsequently promoted cell cycle progression in Hep3B cells. n=three independent repeats, *P<0.05 by ANOVA. (E) The migration and invasion capacities were repressed by miR-376c-3p knockdown, and accordingly reversed by ARID2 silencing in Hep3B cells. n= five filed of three independent repeats, *P<0.05 by ANOVA.

